# Transcriptomic and Network Analysis Identifies Shared and Unique Pathways across Dementia Spectrum Disorders

**DOI:** 10.3390/ijms21062050

**Published:** 2020-03-17

**Authors:** Jose A. Santiago, Virginie Bottero, Judith A. Potashkin

**Affiliations:** 1 NeuroHub Analytics, LLC, Chicago, IL 60605, USA; jose.santiago.ecm@gmail.com; 2 Center for Neurodegenerative Diseases and Therapeutics, Cellular and Molecular Pharmacology Department, The Chicago Medical School, Rosalind Franklin University of Medicine and Science, North Chicago, IL 60064, USA; Virginie.Bottero@rosalindfranklin.edu

**Keywords:** Alzheimer’s disease, vascular dementia, frontotemporal dementia, network and pathway analysis, kruppel-like factor 4

## Abstract

Background: Dementia is a growing public health concern with an estimated prevalence of 50 million people worldwide. Alzheimer’s disease (AD) and vascular and frontotemporal dementias (VaD, FTD), share many clinical, genetical, and pathological features making the diagnosis difficult. Methods: In this study, we compared the transcriptome from the frontal cortex of patients with AD, VaD, and FTD to identify dysregulated pathways. Results: Upregulated genes in AD were enriched in adherens and tight junctions, mitogen-activated protein kinase, and phosphatidylinositol 3-kinase and protein kinase B/Akt signaling pathways, whereas downregulated genes associated with calcium signaling. Upregulated genes in VaD were centered on infectious diseases and nuclear factor kappa beta signaling, whereas downregulated genes are involved in biosynthesis of amino acids and the pentose phosphate pathway. Upregulated genes in FTD were associated with ECM receptor interactions and the lysosome, whereas downregulated genes were involved in glutamatergic synapse and MAPK signaling. The transcription factor KFL4 was shared among the 3 types of dementia. Conclusions: Collectively, we identified similarities and differences in dysregulated pathways and transcription factors among the dementias. The shared pathways and transcription factors may indicate a potential common etiology, whereas the differences may be useful for distinguishing dementias.

## 1. Introduction

Dementia is a major cause of disability and dependency in the elderly with a paramount social and economic impact. The prevalence of dementia is expected to increase to 131 million in the next decades [1]. Early and accurate diagnosis of dementia is a challenging task for clinicians and more than 50% of the cases remain undiagnosed [1]. One of the major hurdles in diagnosing and treating dementia is the presence of clinically and pathologically similar types of dementia. The most prevalent cause of dementia is Alzheimer’s disease (AD) [2]. However, other types of dementia including vascular dementia (VaD) and frontotemporal dementia (FTD), for example, frequently occur and share many clinical and pathological features making the diagnosis difficult. 

AD is characterized by the accumulation of amyloid-beta plaques and protein tau in the form of neurofibrillary tangles. Mutations in the amyloid precursor protein (APP), and presenilin 1 (PSEN1) and presenilin 2 (PSEN2) cause early onset AD. Nonetheless, most of the AD cases are late onset and sporadic, most likely a consequence of complex interactions between genetic and environmental factors. Clinically, AD dementia is defined as the progressive impairment and deterioration of cognition, spatial cognition, episodic memory, and executive functions affecting activities of daily living [3]. There are two different phenotypes in AD dementia, the typical amnestic AD characterized by impairments in recall and learning new information, and the atypical non-amnestic associated with deficits in word-finding, spatial cognition, and executive functions [3]. Accurate assessment of clinical symptoms is the only available tool for distinguishing between these different phenotypes. 

VaD is the second most common cause of dementia after AD, responsible for approximately 15% of the dementia cases. Clinically, VaD is characterized by a rapid onset and stepwise progression of cognitive impairment following a stroke [4]. VaD can result from injuries to the vessels supplying blood to the brain. Indeed, dementia develops in approximately 15%–30% of subjects 3 months after a stroke [4]. Similar to AD, VaD has different causes and clinical presentations. There is a lack of consensus on the definition of VaD due to the prominent overlap in symptoms and risk factors between VaD and AD. Shared risk factors include increasing age, low educational levels, female sex, vascular risk factors, presence of strokes, diabetes, high blood pressure, and hypertension [4]. In addition, the degree of cognitive impairment in VaD is similar to the one observed in AD. A definite diagnosis of VaD requires the presence of significant cerebrovascular disease in addition to the cognitive impairment assessment by clinicians. Brain imaging techniques including computer tomography (CT) scans and magnetic resonance imaging (MRI) have been useful for identifying infarct zones and white matter lesions characteristic of VaD [4]. Because of the substantial overlap between VaD and other dementias, in particular with AD, there is pressing need to distinguish them at the molecular level. 

Frontotemporal lobar dementia (FTD) is a highly heterogenous disease consisting of three genetically, pathologically, and clinically different diseases that affect the temporal and frontal lobes [5]. The three distinct clinical types include the behavioral variant frontotemporal dementia (Bv-FTD), progressive non-fluent aphasia (PNFA), and semantic variant primary progressive aphasia (SV-PPA) [5]. Bv-FTD is the most frequent clinical syndrome of FTD and is characterized by a progressive deterioration of personality and behavior, lack of empathy, apathy, cognitive impairments with relatively no impairment in episodic and semantic memory [5,6]. FTD can be further divided into those individuals with tau positive inclusions FTD-tau and those with ubiquitinated inclusions (FTD-U) [7]. The presence of the RNA binding protein TDP-43 encoded by TARDP is a major pathological hallmark of FTD-U [8,9]. There are no reliable biomarkers for diagnosing FTD patients and therefore, the correct use of clinical criteria is the only accessible tool for clinicians. 

Despite the above discussed pathological and clinical differences, distinguishing between the several types of dementia remains a challenge and a subject of extensive investigation. Indeed, there is no fully validated biomarker with the required sensitivity and specificity for accurately diagnosing patients within the dementia spectrum. Network-based approaches have been useful for revealing some of the mechanistic pathways involved in the development of different dementias including, AD [10,11,12,13,14], VaD [15], and FTD [16,17,18]. Very few studies, however, have employed network-based approaches for identifying unique features that could help distinguishing dementias. In this study, we used network, pathway and transcription factor analyses for identifying genes associated with the transition from a healthy aging brain to a dementia state. 

## 2. Results

### 2.1. Database Mining for Brain Transcriptomic Studies

**Figure 1:**
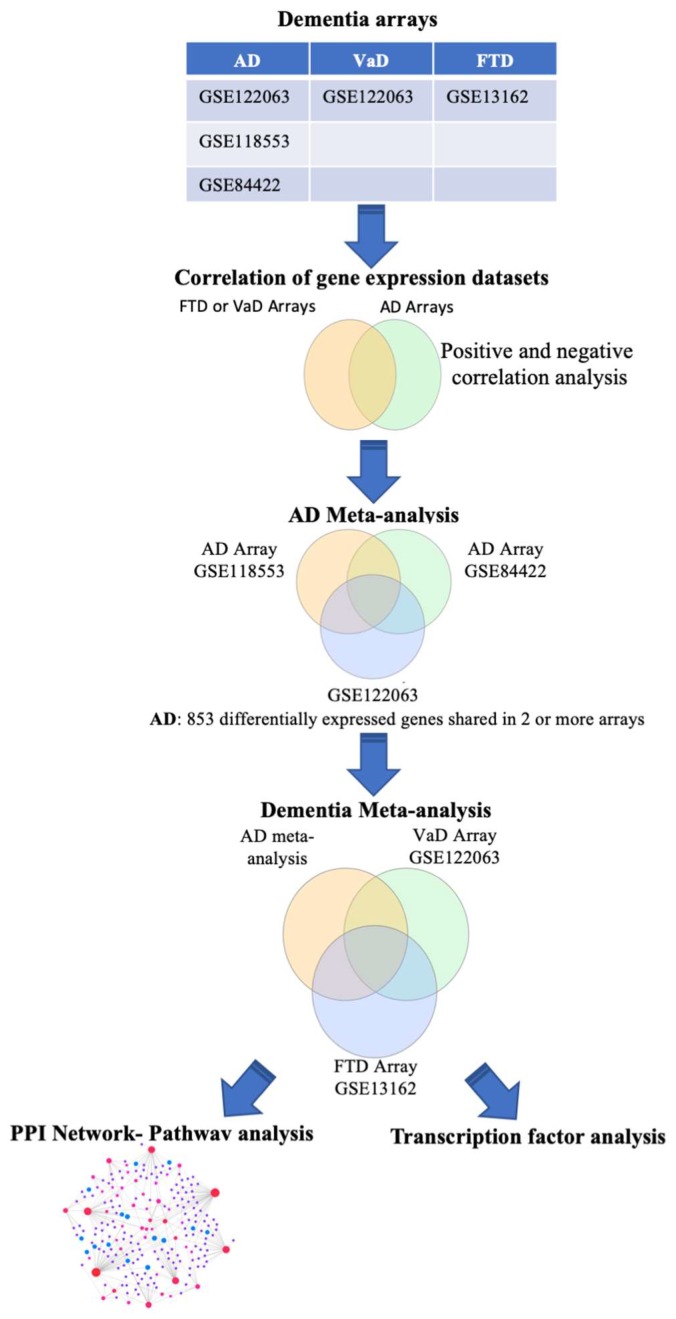
Flowchart of the study. Microarray data from postmortem brain tissue of Alzheimer’s disease (AD), vascular dementia (VaD), and frontotemporal dementia (FTD), patients was curated and differential gene expression analyzed using the BaseSpace Correlation Engine (BSCE). Meta-analysis of 3 independent AD studies was performed using BSCE. The list of genes identified in the AD meta-analysis was compared to the differentially expressed genes in VaD and FTD using a Venn diagram analysis. Protein–protein interactions and transcription factors (TF) network analysis identified dysregulated pathways and TF shared and unique to each dementia.

The overall analysis strategy is presented in Figure 1.

Initially, searching the Array Express and NCBI GEO databases, we identified studies that contained expression data from postmortem brain tissue of AD, VaD, and FTD patients and age-matched controls. Four independent studies that met our inclusion criteria (See Methods) were identified containing transcriptomic data from frontal cortex (GSE122063, GSE11853, GSE84422, and GSE13162). GSE122063 dataset contained data from both AD and VaD individuals. Description of the datasets analyzed in this study is presented in Table 1. 

### 2.2. Correlation Analysis of Gene Expression Datasets from AD, VaD, and FTD Subjects

In order to compare the gene expression patterns of individuals with AD with those with VaD and FTD, we performed a correlation analysis using BSCE. We compared the numbers of shared differentially expressed genes as well as the directionality of the fold changes. Gene expression profiles of individuals with FTD significantly overlapped and correlated positively with those from individuals with AD in 2 out of the 3 studies (Figure S1a–c). Similarly, gene expression profiles of individuals with VaD significantly overlapped and correlated positively with those from individuals with AD in all 3 studies (Figure S1d–f), as did the expression profiles from individuals with VaD and FTD. Correlation analysis of VaD and FTD showed both positive and negative correlations (Figure S1g). 

### 2.3. Analysis of Differentially Expressed Genes in AD, VaD, and FTD Individuals

In order to identify consensus among the different transcriptomic datasets from AD patients, we performed a meta-analysis using BSCE. Meta-analysis of the 3 AD microarrays was performed using only genes that were dysregulated in the same direction in at least 2 out of 3 arrays with a fold-change of 1.2 or more. This analysis resulted in 282 upregulated and 571 downregulated transcripts in the frontal cortex of AD patients compared to non-demented controls (Table 2 and Table S1). The most significant gene identified in the meta-analysis was aquaporin 1 (AQP1). AQP1 was upregulated in AD subjects compared to non-demented controls in 2 out the 3 AD studies (Table S1). 

We next compared the set of differentially expressed genes identified in the meta-analysis from AD subjects to those genes identified in VaD and FTD. Analysis of differentially expressed genes using BSCE identified 528 upregulated and 622 downregulated genes in the frontal cortex of patients with VaD compared to controls (Table 2 and Table S2). Similarly, we identified 437 upregulated and 132 downregulated genes in the frontal cortex of FTD patients compared to controls (Table 2 and Table S3). 

**Figure 2:**
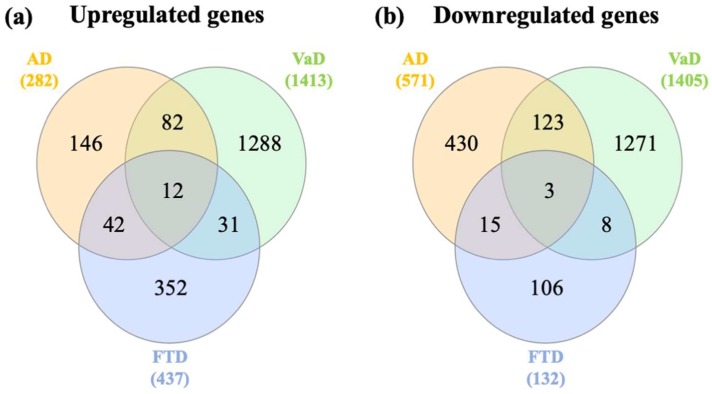
Venn diagram analysis of differentially expressed genes. (**a**) Venn diagram analysis of upregulated genes in AD, VaD, and FTD. (**b**) Venn diagram analysis of downregulated genes in AD, VaD, and FTD. The genes in the AD group represent the genes identified in the meta-analysis of AD datasets.

Venn diagram analysis was performed to determine the shared and unique genes between the 3 dementia (Table S4). Venn analysis of upregulated genes identified 12 genes including *AQP1*, microtubule associated scaffold protein 1 (*MTUS1*), ras homolog family member Q (*RHOQ*), CASP8 and FADD like apoptosis regulator (*CFLAR*), KH domain containing RNA binding (*QK1*), solute carrier family 38 member A2 (*SLC38A2*), mitogen-activated protein kinase kinase kinase kinase 5 (*MAP4K5*), BBX high mobility group box domain containing (BBX), interleukin 6 signal transducer (*IL6ST*), CD164 molecule (*CD164*), ST13 Hsp70 interacting protein (*ST13*), TGF-beta activated kinase 1 (*MAP3K7*) binding protein 2 (*TAB2*) shared between AD, VaD and FTD (Figure 2a). Likewise, Venn analysis of downregulated genes identified 3 genes, dual specificity phosphatase 6 (*DUSP6*), *VGF* nerve growth factor inducible (*VGF*), and activity regulated cytoskeleton associated protein (*ARC*) shared between dementia types (Figure 2b).

### 2.4. Shared and Unique Biological Pathways in AD, VaD, and FTD

In order to understand the functional role of differentially expressed genes we performed a network and pathway analysis for each gene set corresponding to AD, VaD, and FTD. Upregulated and downregulated gene sets were analyzed separately using NetworkAnalyst. Tissue specific networks derived from the protein-protein interaction database from human frontal cortex were constructed for each disease. The minimum connected network was selected for further pathway analysis. 

**Figure 3:**
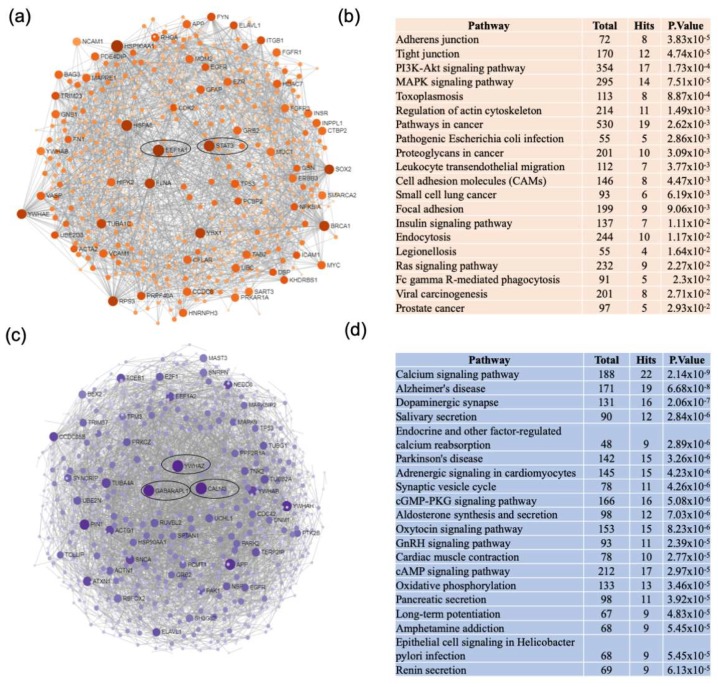
Network and pathway analyses of dysregulated genes in the frontal cortex of AD patients. Integrative meta-analysis was performed on datasets from AD patients compared to non-demented controls to identify unique dysregulated pathways in the frontal cortex of AD. Network and pathway analyses were performed using NetworkAnalyst. Tissue specific networks derived from the protein-protein interaction database from brain frontal cortex were selected. The minimum connected network was analyzed further. Results from the pathway analysis are derived from the Kyoto Encyclopedia of Genes and Genome (KEGG). (**a**) Upregulated genes are shown in orange. Most significant hubs according to degree and betweenness centrality, with the highest number of connections, are enclosed in ovals. (**b**) The 20 top dysregulated pathways ranked according the p-value of significance are presented in the table. Totals refers to the total number of genes that are known to be involved in the KEGG pathway and hits refers to the number of genes identified in this analysis that are involved in the pathway. (**c**) Downregulated genes are shown in blue. Most significant hubs according to degree and betweenness centrality, with the highest number of connections, are enclosed in ovals. (**d**) The 20 top dysregulated pathways ranked according the p-value of significance are presented in the table.

Network analysis of upregulated genes identified in the meta-analysis of AD datasets resulted in a unique network centered on signal transducer and activator of transcription 3 (STAT3) and elongation factor 1 alpha (EEF1A). This network was predominantly enriched in genes associated with adherens and tight junctions, mitogen-activated protein kinase (MAPK), and phosphatidylinositol 3-kinase and protein kinase B/Akt (PI3K-AKT) signaling pathways (Figure 3a,b). Analysis of downregulated genes identified in the meta-analysis of AD datasets resulted in a unique network centered on calmodulin 3 (CALM3). Downregulated genes were predominantly enriched in the calcium signaling pathway (Figure 3c,d). 

**Figure 4:**
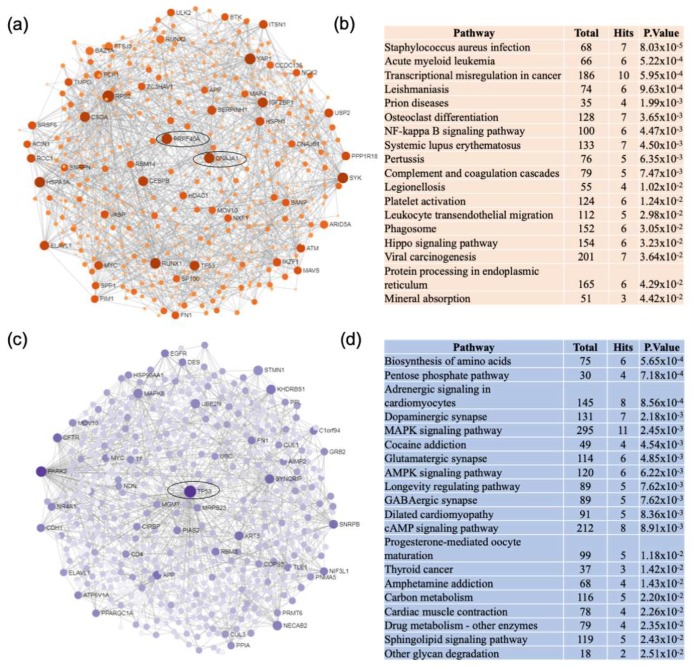
Network and pathway analyses of dysregulated genes in the frontal cortex of VaD patients. Network and pathway were performed on the dataset from VaD patients compared to non-demented controls to identify unique dysregulated pathways in the frontal cortex of VaD. Network and pathway analyses were performed using NetworkAnalyst. Tissue specific networks derived from the protein-protein interaction database from brain frontal cortex were selected. The minimum connected network was analyzed further. Results from the pathway analysis are derived from the Kyoto Encyclopedia of Genes and Genome (KEGG). (**a**) Upregulated genes are shown in orange. Most significant hubs according to degree and betweenness centrality, with the highest number of connections, are enclosed in ovals. (**b**) The 20 top dysregulated pathways ranked according the p-value of significance are presented in the table. Totals refers to the total number of genes that are known to be involved in the KEGG pathway and hits refers to the number of genes identified in this analysis that are involved in the pathway. (**c**) Downregulated genes are shown in blue. Most significant hubs according to degree and betweenness centrality, with the highest number of connections, are enclosed in ovals. (**d**) The 20 top dysregulated pathways ranked according the p-value of significance are presented in the table.

The same network analysis was performed for the VaD dataset. The unique network of upregulated genes in VaD was centered on DnaJ heat shock protein family (DNAJA1) and pre-mRNA processing factor 40 homolog A (PRPF40A) and were enriched predominantly in infectious diseases and inflammatory pathways (Figure 4a,b). Interesting upregulated pathways included nuclear factor kappa beta (NF-κB) signaling, complement and coagulation cascade, and protein processing in the endoplasmic reticulum. Downregulated genes formed a network centered on tumor protein p53 (TP53) and were enriched in biosynthesis of amino acids, pentose phosphate pathway, and adrenergic signaling in cardiomyocytes (Figure 4c,d). Other cardiovascular related pathways were identified including cardiac muscle contraction and hypertrophic cardiomyopathy. 

**Figure 5:**
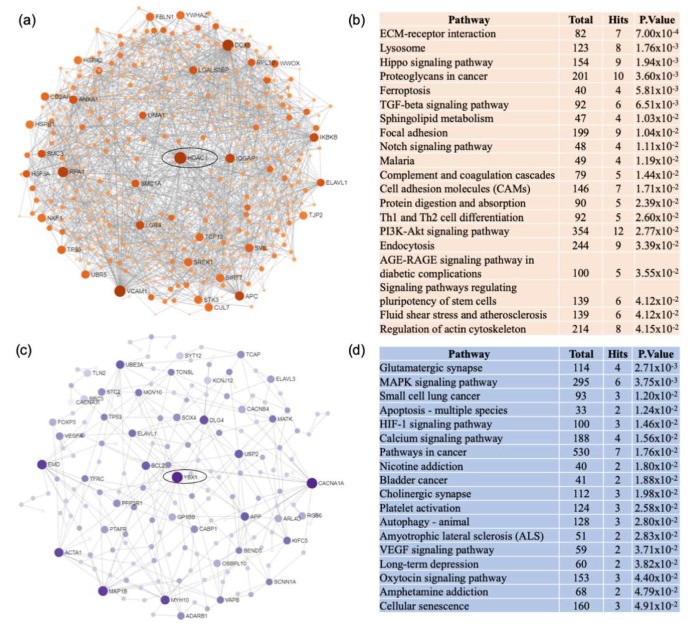
Network and pathway analyses of dysregulated genes in the frontal cortex of FTD patients. Network and pathway were performed on the dataset from FTD patients compared to non-demented controls to identify unique dysregulated pathways in the frontal cortex of FTD. Network and pathway analyses were performed using NetworkAnalyst. Tissue specific networks derived from the protein-protein interaction database from brain frontal cortex were selected. The minimum connected network was analyzed further. Results from the pathway analysis are derived from the Kyoto Encyclopedia of Genes and Genome (KEGG). (**a**) Upregulated genes are shown in orange. Most significant hubs according to degree and betweenness centrality, with the highest number of connections, are enclosed in ovals. (**b**) The 20 top dysregulated pathways ranked according the p-value of significance are presented in the table. Totals refers to the total number of genes that are known to be involved in the KEGG pathway and hits refers to the number of genes identified in this analysis that are involved in the pathway. (**c**) Downregulated genes are shown in blue. Most significant hubs according to degree and betweenness centrality, with the highest number of connections, are enclosed in ovals. (**d**) The 20 top dysregulated pathways ranked according the p-value of significance are presented in the table.

Analysis of upregulated genes in FTD resulted in a unique network centered on histone deacetylase 1 (HDAC1) and were enriched in pathways related to ECM-receptor interaction, lysosome, and hippo signaling pathways (Figure 5a,b). Notably, PI3K-AKT signaling pathway was also upregulated but with a lesser significance than in AD. Network analysis of unique downregulated genes in FTD identified a network centered on Y box binding protein 1 (YBX1) and enriched predominantly in glutamatergic synapse and MAPK signaling pathways (Figure 5c,d). Similar to AD, calcium signaling pathway was downregulated in FTD.

We next determined shared and unique pathways between each dementia (Table S5). Venn diagram analysis showed that 4 pathways, MAPK signaling, glutamatergic synapse, amphetamine addiction, and platelet activation were shared between the 3 types of dementia. In addition, Venn analysis indicated that AD shared 16 pathways with VaD and 19 pathways with FTD, whereas VAD and FTD shared 2 pathways. The total number of unique pathways for AD, VaD, and FTD were 65, 21, and 15, respectively.

### 2.5. Gene-Transcription Factors Interaction Analysis

**Figure 6:**
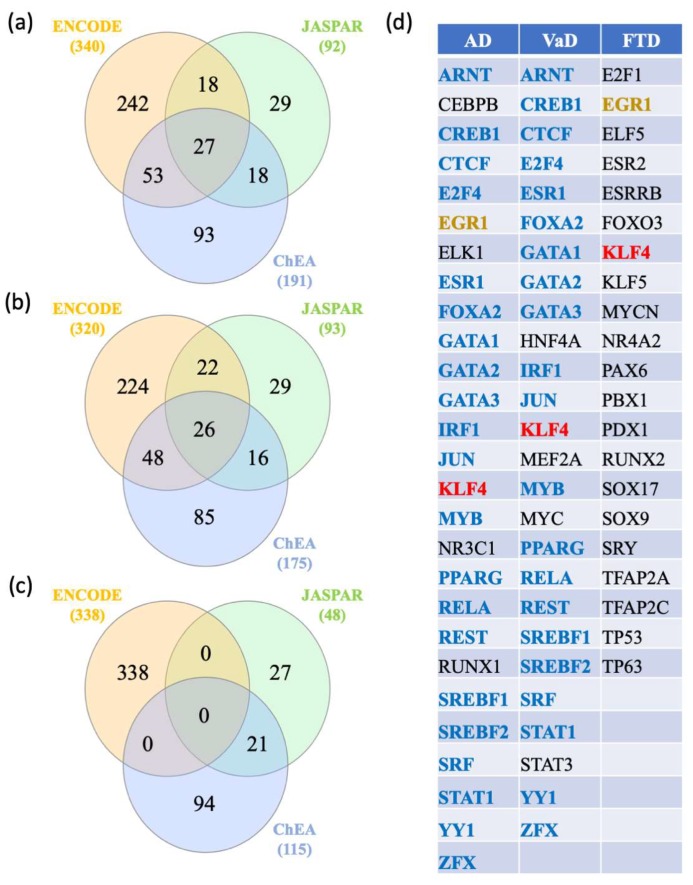
Transcription factors analysis. The AD, VaD, and FTD genes lists were uploaded to NetworkAnalyst, https://www.networkanalyst.ca/NetworkAnalyst/faces/home.xhtml. The gene-transcription factor interaction network was performed using ENCODE, ChEA, and JASPAR databases and (**a**), (**b**), and (**c**) represent the results of the Venn diagram analysis performed with AD, VaD and FTD genes, respectively. The transcription factors interacting with the AD, VaD, and FTD genes were listed in (**d**). The transcription factor in red is shared between the 3 types of dementia. The transcription factors in blue are shared between AD and VaD. The transcription factor in brown is shared between AD and FTD. The transcription factors in black are unique to each dementia analysis.

In order to identify key regulators of the AD, VaD, and FTD dysregulated genes, a transcription factor analysis was performed using the ENCODE, ChEA, and JASPAR databases and transcription factors that were shared were identified by Venn diagram analysis (Figure 6a,b,c). In the AD analysis, 27 transcription factors were shared by all the databases (Figure 6a,d) whereas 26 transcription factors were identified in the VaD analysis (Figure 6b,d). In the FTD analysis, no shared transcription factors were found between the 3 databases. Using ENCODE and ChEA did not identified any shared transcription factors. However, 21 transcription factors were identified shared between JASPAR and ChEA databases (Figure 6c,d). We next investigated shared transcription factors among the 3 types of dementia (Figure 6d). Venn diagram analysis showed that AD and VaD shared 21 transcription factors (IRF1, ZFX, REST, CTCF, PPARG, CREB1, YY1, ARNT, STAT1, GATA1, GATA2, GATA3, SREBF1, SREBF2, RELA, FOXA2, MYB, E2F4, JUN, SRF, ESR1) whereas AD and FTD shared one transcription factor, EGR1. Finally, Venn analysis identified kruppel-like factor 4 (KLF4) as the only transcription factor shared between the 3 dementias. 

## 3. Discussion

In the last decade there has been a rising interest in differentiating the various forms of dementia including AD, VaD, and FTD. Although good progress has been made in understanding the genetic basis and identifying some of the biological mechanisms involved in dementia, the path towards personalized treatment remains unclear. 

We first compared the brain transcriptome of AD patients to those affected by VaD and FTD. We focused the analysis on the frontal cortex since this brain region is commonly affected in dementia and neurodegenerative diseases [19,22,23]. Correlation analyses showed that gene expression profiles of subjects with AD significantly overlapped and positively correlated with those affected by VaD and FTD suggesting that similar molecular changes occur in the frontal cortex of these dementia types. 

In order to identify a common set of differentially expressed genes in AD, we performed a meta-analysis of 3 independent datasets from AD subjects. The most significant gene identified in the meta-analysis was AQP1. AQP1 is a membrane water channel protein, which under normal physiological conditions is expressed mainly in the choroid plexus epithelium that is located within the cerebral ventricles and forms the blood and cerebrospinal fluid barrier. AQP1 is involved in CSF formation [24] and may serve as an osmosensor [25]. Interestingly, increased expression of AQP1 has been found in reactive astrocytes of several neurodegenerative diseases including Creutzfeldt-Jakob disease, multiple sclerosis, and AD. In AD brains, reactive astrocytes expressing AQP1 were found in close proximity to amyloid beta (Aβ) plaques suggesting a possible role of astrocytic AQP1 in the deposition of Aβ [26]. Another study found increased expression of AQP1 in astrocytes in early stage (Braak II) AD patients [27]. Consistent with these findings, the results from our meta-analysis showed that AQP1 is upregulated in the frontal cortex of AD patients compared to non-demented controls. Thus, increased AQP1 expression in astrocytes may be indicative of alterations in the control of water fluxes at early stages of AD. 

In addition to AQP1, we identified another 11 upregulated genes shared between AD, VaD, and FTD that have been associated with neurodegeneration. For instance, *MTUS1* expression correlated with Braak staging in AD patients and it may be associated to changes in hippocampal volume prior to onset of cognitive impairment [28]. Similarly, *QK1*, a gene exclusively expressed in glial cells, was upregulated in human postmortem brain samples from AD patients compared to healthy controls [29]. Expression levels of *QK1* and *QK1* isoforms were predictive of the variation of expression of AD-related genes *APP, PSEN1, PSEN2,* and *MAPT* [29]. 

Analysis of downregulated genes identified 3 genes shared between AD, VaD, and FTD. One example, VGF, has been widely studied in several neurodegenerative diseases. Most of the studies on VGF are biomarker studies. For example, VGF levels in cerebrospinal fluid (CSF) were lower in dementia with Lewy bodies compared to AD and healthy controls [30]. Similarly, FTD patients carrying a progranulin mutation had lower CSF levels of VGF compared to pre-symptomatic carriers and non-carriers [31]. Furthermore, VGF levels were lower in CSF of AD patients compared to controls and outperformed other biomarkers including CSF Aβ1-42, phosphorylated tau, and hippocampal volume in predicting MCI to AD conversion [32]. Replication of these biomarkers in a larger cohort of patients including different types of dementia will be important to assess their diagnostic value. 

We next performed a network and pathway-based analysis to identify shared and unique biological pathways associated with the different dementia types. This analysis was performed using the sets of differentially expressed genes identified for each dementia. Upregulated and downregulated genes were analyzed independently for each condition. Network and pathway analysis of upregulated genes in AD revealed a network enriched in pathways associated with adherens and tight junctions, MAPK, and PI3K-AKT signaling. One of the main gene hubs in the upregulated network was STAT3, a key transcriptional regulator of reactive astrogliosis [33]. In this regard, pharmacological targeting of reactive astrogliosis pathway has shown promise in promoting cell survival and neuroprotection in several neurodegenerative diseases including AD. For example, deletion of STAT3 in astrocytes in the APP/PS1 model of AD decreased beta amyloid plaque formation and ameliorated spatial learning and cognitive decline [34]. Interestingly, STAT3 has been shown to promote the activation of sphingosine kinases and the production of sphingosine 1 phosphate (S1P) in inflammation related pathways in cancer [35]. In the context of AD, the loss of sphingosine kinase 2 activity and SP1 production are key pathogenic drivers of Aβ-mediated neurodegeneration [36]. Another upregulated pathway of interest is PI3K-AKT signaling, which has been extensively implicated in the pathogenesis of AD given its crucial role mediating insulin effects in the brain and other functions in microglia and astrocytes [37]. Collectively, our results from the network analysis support STAT3 as an important transcriptional regulator in AD. Its potential involvement in PI3K-AKT and neuroinflammation warrants further investigation. 

During normal aging neurons may lose the ability to regulate calcium. The increased intracellular calcium levels can become toxic to the cells and initiate the neurodegenerative process. The calcium hypothesis in AD is well documented and drugs targeting calcium channels have been suggested as potential therapeutics in AD. In support of this hypothesis, network analysis of downregulated genes in the frontal cortex of AD subjects revealed a network centered on CALM3 and predominantly enriched in calcium signaling pathway. CALM3 is one of three genes encoding calmodulin protein, which is a calcium sensing and signal transducer protein that modulates several calcium ion channels [38,39]. Although alterations in CALM3 have not yet been documented in AD, given its functional role in calcium signaling, it is reasonable to hypothesize that it may be involved in calcium dysregulation in AD patients. Future studies investigating the functional role of CALM3 will be crucial to understand its implications in AD. 

In contrast to AD, network analysis of upregulated genes in VaD revealed a network enriched in inflammatory pathways, infectious diseases, and protein processing in the endoplasmic reticulum. The resulting network was centered on DNAJA1 and PRPF40A. Among these two genes, DNAJA1 has been implicated in several neurodegenerative diseases including AD and Parkinson’s disease, however, its most significant functional role appears to be the regulation of tau. Specifically, DNAJA1 enhanced ubiquitin mediated proteolysis of mutant tau in the absence of HSP70 in HeLa cell lines [40]. The specific role of DNAJA1 in VaD merits further investigation. The network of downregulated genes in VaD was centered on TP53 and enriched in genes associated to the biosynthesis of amino acids, pentose phosphate pathway, adrenergic signaling in cardiomyocytes and insulin secretion. TP53 has been widely connected to many metabolic pathways including glycolysis, gluconeogenesis, insulin, lipogenesis and oxidative phosphorylation. Among these pathways, the pentose phosphate pathway plays a key role in the defense mechanism against reactive oxygen species (ROS) and it is intimately related to glucose metabolism in the brain. The pentose phosphate pathway is essential for the production of NADPH and maintenance of glutathione in the reduced form (GSH) in order to detoxify ROS. It is well established that hyperglycemia promotes inflammation and the production of ROS leading to vascular damage [41]. Therefore, dysregulation of the pentose phosphate pathway is likely to exacerbate the insults to the brain vasculature by leaving the brain vulnerable to the toxic ROS effects. Although only a handful of studies have linked the pentose phosphate pathway in dementia [42,43], targeting this pathway could be a novel route for therapeutic intervention in VaD. 

Network analysis of upregulated genes in FTD associated with ECM-receptor interaction and the lysosome. These results confirm previous studies that have identified the disruption of the lysosome/endosome system in FTD [44]. Dysfunction of the lysosome/endosome system is responsible for the failure to clear toxic TDP-43 protein aggregates which eventually result in neuronal cell death. 

Shared gene expression patterns between different dementia types may indicate common mechanistic pathways. Four pathways including MAPK signaling, glutamatergic synapse, amphetamine addiction, and platelet activation were shared between AD, VaD, and FTD. From these pathways, MAPK signaling was among the top ranked pathways identified for each dementia. Interestingly, genes associated with MAPK signaling were upregulated in AD but downregulated in VaD and FTD indicating an inverse association at the pathway level between these diseases. Dysregulation in MAPK signaling has been shown to contribute to the pathogenesis of many neurodegenerative diseases including AD, PD, and amyotrophic lateral sclerosis [45]. This is not surprising since protein kinases in this pathway regulate many cellular activities including cellular differentiation, proliferation, apoptosis, inflammation, and innate immunity [45]. In AD, the crosstalk between insulin and MAPK signaling pathways may play a role in the disease pathogenesis [37,46]. Similar pathways crosstalk may be involved in the pathogenesis of FTD. For instance, ubiquilin 2 (UBQLN2), which plays a role in the ubiquitin proteasome system and mutations are associated with FTD, enhanced the activation of NFKB activity through MAPK signaling resulting in TDP-43 aggregation in vitro [47]. In the context of VaD, a MAPK inhibitor reduced apoptosis and rescued memory deficits in a rat model of VaD [48]. Overall, these findings highlight the diverse routes in which alterations in MAPK signaling may contribute to the pathogenesis of different types of dementia. 

Analysis of shared pathways between AD and VaD identified 16 pathways in common. Interestingly, among these pathways, insulin secretion, carbon metabolism, and sphingolipid metabolism were downregulated suggesting the impact of common metabolic impairments in these diseases. The role of both insulin and sphingolipids is well documented in AD and other neurodegenerative diseases [49,50]. Impaired insulin signaling and diabetes have been extensively implicated in the pathogenesis of AD [13,37]. Sphingolipids are key components of cell membranes and play a pivotal role in mediating neuroinflammation, a central pathway involved in the pathogenesis of AD. Disruption of sphingolipid metabolism in the brain has been suggested to promote aberrant amyloid processing and synaptic dysfunction in AD [51]. In the context of VaD, the role of lipid metabolism remains poorly understood and studies addressing this important pathway are lacking. One study that analyzed the lipidome profile from the temporal cortex of subjects with ischemic VaD revealed that sphingolipid alterations disrupted the structure of myelin, an important component for neural conduction [52]. More studies investigating the involvement of lipids in the pathogenesis of VaD are needed to confirm these results. 

Similarly, AD and FTD shared 19 pathways. Notably, PI3K-AKT and calcium signaling pathways were upregulated and downregulated, respectively, in AD and FTD. While dysregulation of calcium is well documented in AD, studies addressing this pathway in FTD are scarce. A network-based approach suggested that impairment in calcium/cAMP and energetic metabolism are the primary causative factors in the neuronal cell death in FTD [53]. The impairment of these pathways was observed particularly in astrocytes, suggesting that the loss of calcium/cAMP homeostasis may be involved in the progressive neurodegeneration in FTD. 

Analysis of transcription factors identified KLF4 as a common regulator in AD, VaD and FTD. The role of KLF4 as potential therapeutic target in AD has been proposed [54]. Silencing of KLF4 attenuated the release of proinflammatory cytokines caused by accumulation of Aβ42 oligomers in a cellular model of AD [55]. Furthermore, KLF4 has been implicated in an epigenetic mechanism in AD. Briefly, hypermethylation of KLF4 and TRIM59 associated with alterations in DNA repair and cell cycle control suggesting these genes may be involved in cellular apoptosis in AD [56]. To the best of our knowledge, there is no evidence for the involvement of KLF4 in VaD and FTD. Nonetheless, given the involvement of KLF4 in apoptosis and neuroinflammation, future studies investigating its potential therapeutic value are warranted. 

Our results are based on publicly available microarray data and therefore several limitations to this study should be noted. The power of the analysis may be influenced by sample size and in this study, sample size was small despite the fact that we selected all microarray data sets that have more than 5 patients and controls. The data was curated using the database BaseSpace Correlation Engine for quality and normalization of the data. Genes whose mean normalized test and control intensities were both less than the 20^th^ percentile of the combined normalized signal intensities were removed. Because of the limited data that is currently available, it will be important to replicate our results in larger studies. 

Another limitation is site-to-site variability. The selected studies were performed in different laboratories and therefore, differences in the diagnostic, sample collection methods or sample preparation might influence the results. An additional difference between the different sites was the information provided on the study participants. For the AD analysis, the patient information was limited to age, postmortem interval and gender. Diagnosis of AD was based on cognitive testing and BRAAK pathology scores were provided to characterize disease progression. Unfortunately, additional information such as the presence of pathogenic mutations (APP, PSEN1, and PSEN2) as well as the ApoE status of the participants were not available. In the VaD analysis, the patients were diagnosed with multi-infarct dementia, where multiple areas of the brain have been injured due to a lack of blood from a series of small strokes. These patients had no evidence, or very minimal evidence of, any AD typical pathology. In the FTD analysis, we selected microarrays containing patients with no GRN mutations, however information on MAPT mutations and C9orf72 repeat expansion was not available. It is possible, however, that the participants in all of the studies used in our analysis may carry an unknown genetic mutation that affects dementia. Additional gene expression data from participants in studies that include genetic information will be helpful in revealing genetic loci that influence gene expression.

## 4. Methods

### 4.1. Database Mining

The NCBI GEO database (https://www.ncbi.nlm.nih.gov/gds) and ArrayExpress database (https://www.ebi.ac.uk/arrayexpress/) were searched on October 1, 2019 for studies in which transcriptomic data was available from frontal cortex in the brain of individuals with AD, VaD, FTD. Using the search terms “Alzheimer’s disease”, “vascular dementia”, “frontotemporal dementia”, “brain”, “frontal cortex”, “human” “RNA”, and “microarray”, we identified 18 brain transcriptomic studies. Only human transcriptomic studies from frontal cortex brain region and with more than 5 cases and controls were included for further analysis. In addition, studies in which participants had known genetic mutations related to dementia were not included because we wanted to focus this study on understanding sporadic forms of dementia. We also excluded studies that included individuals with diabetes, an established comorbidity of dementia. Four microarrays met our inclusion criteria (Table 1). 

The microarrays were curated using the database BaseSpace Correlation Engine (BSCE, Illumina, Inc., San Diego, CA, USA). Individuals who carried known genetic mutations were not included in the final analysis. 

### 4.2. Clinical and Demographic Characteristics of Participants Included in the Study

The characteristics of the samples analyzed in this paper are presented in Table 3.

In this study we analyzed 3 independent microarrays from AD patients. In the dataset GSE84422, the original study design included 125 human brains from the Mount Sinai/JJ Peters VA Medical Center Brain Bank (MSBB). From this large dataset, we analyzed a subset comprising 21 samples from the prefrontal cortex of patients with definite AD compared to 11 normal controls. Patients with neuropathological lesions not associated with AD, multi-infarct dementia, and cerebrovascular disease were excluded [57]. The dementia severity was measured on the Clinical Dementia Rating scale [58]. 

For the dataset GSE118553, brain samples were obtained from the Medical Research Council of London Neurodegenerative Diseases Brain Bank (MRC-LBB). Informed consent was obtained from all the subjects. A subset of 52 AD cases with a clinical diagnosis of AD at death and neuropathological evaluation at autopsy, were selected for the analysis. Braak stage for AD patients was 4.9 ± 1. The control group included 27 cases and did not present any clinical signs of dementia or neuropathology. A detailed description of the subjects analyzed in this study are described in [19].

The dataset GSE122063 included samples from both AD and VaD subjects. Brain samples were collected from 12 AD, 9 VaD, and 10 age, postmortem interval, and gender matched controls from the University of Michigan Alzheimer’s Disease Center Brain Bank. The AD patients were classified within the Braak stage III-IV. VaD samples were obtained from subjects with multi-infarct dementia subtype. AD patients and VaD patients had no infarcts present in the autopsied hemisphere and no evidence or very minimal evidence of any AD typical pathology, respectively. This pathological confirmation allowed for exclusion of likely mixed dementia cases, which could bias the results. Demographic and clinical characteristics about this cohort of patients is described in [15]. 

For the dataset GSE13162 from FTD patients, we analyzed the samples from individuals classified as sporadic FTD by a board-certified neuropathologist [21]. The FTD patients included in our analysis had ubiquitinated inclusions but no mutations in the progranulin gene (GRN). The dataset included 10 FTD-U cases and 8 neurologically normal age, sex and postmortem interval matched controls from the University of Pennsylvania Center for Neurodegenerative Disease Research Brain Bank. 

### 4.3. Transcriptomic Analysis of Gene Expression Datasets from AD, VaD, and FTD Individuals

For every two datasets, the genetic overlap among the different gene expression datasets was analyzed as previously described [13,59]. Briefly, BSCE used a ‘Running Fisher’ algorithm to overlap the p values between different gene expression datasets [59]. A p-value of 0.05 or less was considered significant. Microarray meta-analyses of the AD datasets were performed in BSCE as described previously [13,60]. The Venn diagrams and the correlation graphs were obtained from BSCE. Differentially expressed genes were extracted from BSCE. If any negative values were present, they were replaced with the smallest positive number in the dataset. Genes whose mean normalized test and control intensities were both less than the 20th percentile of the combined normalized signal intensities were removed. The meta-analysis tool in BSCE used a normalized ranking approach, allowing the elimination of any potential biases introduced by the use of different array platforms or the sample size [59]. The activity of the gene in each dataset and the number of datasets in which the gene is differentially expressed was used to determine the scoring and ranking of each gene. The analysis included only genes with an absolute fold-change of 1.2 or greater and a p-value of 0.05 or less. 

### 4.4. Network and Pathway Analysis 

Entrez gene identifiers from the genes identified in the differential gene expression analysis and meta-analyses were imported into NetworkAnalyst for network and pathway analyses [61]. NetworkAnalyst can be access via the webpage https://www.networkanalyst.ca/. Protein-protein interaction data from frontal cortex brain region were obtained from DifferentialNet (http://netbio.bgu.ac.il/diffnet/) and used to create the tissue specific networks [62]. The minimum connected network was selected for further pathway analysis. We used the pathway analysis data derived from the Kyoto Encyclopedia of Genes and Genome (KEGG). 

### 4.5. Gene-Transcription Factors Interaction Analysis

Entrez gene identifiers from the dyregulated genes were imported into NetworkAnalyst for network analysis of transcription factors. Three transcription factor and gene target databases were used [63,64,65]. In the Encyclopedia of DNA Elements (ENCODE) database, the transcription factors, identified using the BETA Minus Algorithm, have a peak intensity signal < 500 and the predicted regulatory potential score <1. The ChIP Enrichment Analysis (ChEA) transcription factor targets database is based on published ChIP-X data. Finally, JASPAR database includes curated, non-redundant transcription factor (TF)-binding profiles. The network topology measurements, such as degree and betweenness centrality, was used to rank the identified transcription factors. A Venn diagram analysis was performed with the transcription factors identified with each database. 

## 5. Conclusions

Collectively, the results presented in this study suggest that AD, VaD, and FTD may develop from alterations in several unique dysregulated pathways. For example, dysregulation of the PI3-AKT and calcium signaling are more significant to AD whereas inflammation and the pentose phosphate pathway are more predominant in VaD. In contrast, FTD is characterized by alterations in the endosome/lysosome system and alterations in the ECM-receptor interactions. Further investigation on shared metabolic impairments including insulin signaling, sphingolipids and carbon metabolism will be important to better distinguish AD and VaD. In addition, disruption of calcium homeostasis observed in AD and FTD warrants further investigation. Given the involvement of KLF4 in apoptosis and neuroinflammation, follow up mechanistic studies evaluating its potential as a therapeutic target for dementia will be valuable. 

The findings reported here should be taken with caution as they are mostly based on bioinformatic and correlational analyses. Future studies using samples from patients when they become available will be beneficial in validating the results presented in this study. Other possible confounds including differences in microarray platforms, blood collection, and RNA extraction methods; and different clinical criteria used to define patient populations may bias the results. Although mixed pathologies were not reported in any of the studies analyzed, potential disease comorbidities and medication use could impact the results. For these reasons, it is important to confirm the present results by independent analysis. In addition, other tissue samples could be studied to test the generality of this data. In the regard, the choroid plexus transcriptome from AD and FTD patients was recently documented [66]. In addition, other brain tissues could be considered for analysis of AD patients such as the hippocampus [67]. 

## Figures and Tables

**Table 1:** Gene expression datasets selected in this study.

Dataset	Phenotype Type	Brain Region	Platform	Reference
**GSE122063**	Alzheimer’s disease	Frontal cortex	Agilent Human 8x60k v2 microarrays	[15]
**GSE118553**	Alzheimer’s disease	Frontal cortex	Illumina HumanHT-12 V4.0 expression beadchip	[19]
**GSE84422**	Alzheimer’s disease	Frontal cortex	Affymetrix GeneChip Human HG_U133 Plus 2.0	[20]
**GSE122063**	Vascular dementia	Frontal cortex	Agilent Human 8x60k v2 microarrays	[15]
**GSE13162**	Frontotemporal dementia	Frontal cortex	Affymetrix GeneChip Human HG_U133A version	[21]

**Table 2:** The 5 most significant downregulated and upregulated genes for each dementia. Disease associations were obtained from GeneCards website (https://www.genecards.org/).

Dementia	Gene Symbol	Gene Name	Diseases Associated
**AD**			
Down regulated	*SST*	Somatostatin	Somatostatinoma and Esophageal Varix
	*VGF*	VGF Nerve Growth Factor Inducible	Pulmonary Large Cell Neuroendocrine Carcinoma and Vaccinia
	*MAL2*	Mal, T Cell Differentiation Protein 2	Chromophobe Renal Cell Carcinoma
	*SVOP*	SV2 Related Protein	Intestinal Botulism and Familial Atrial Fibrillation
	*BEX5*	Brain Expressed X-Linked 5	
Up regulated	*AQP1*	Aquaporin 1	Blood Group, Colton System and Diabetes Insipidus, Nephrogenic, Autosomal
	*AQP4*	Aquaporin 4	Brain Edema and Neuromyelitis Optica
	*ANGPT2*	Angiopoietin 2	Placental Insufficiency and Macular Holes
	*RHOBTB3*	Rho Related BTB Domain Containing 3	
	*MTUS1*	Microtubule Associated Scaffold Protein 1	Hepatocellular Carcinoma and Temporal Arteritis
**VaD**			
Down regulated	*RBM3*	RNA Binding Motif Protein 3	Testicular Malignant Germ Cell Cancer and Noonan Syndrome 1
	*SSX3*	SSX Family Member 3	Sarcoma, Synovial and Sarcoma
	*GPR45*	G Protein-Coupled Receptor 45	
	*OR6C74*	Olfactory Receptor Family 6 Subfamily C Member 74	
	*GUCY2GP*	Guanylate Cyclase 2G, Pseudogene	
Up regulated	*FCGBP*	Fc Fragment Of IgG Binding Protein	Congenital Hypogammaglobulinemia and Von Willebrand Disease, Type 2
	*AQP1*	Aquaporin 1	Blood Group, Colton System and Diabetes Insipidus, Nephrogenic, Autosomal
	*SNX31*	Sorting Nexin 31	Melanoma, Cutaneous Malignant 1
	*SIGLEC14*	Sialic Acid Binding Ig Like Lectin 14	
	*MIA*	MIA SH3 Domain Containing	Melanoma and Skin Melanoma
**FTD**			
Down regulated	*NPTX2*	Neuronal Pentraxin 2	Narcolepsy and Kearns-Sayre Syndrome
	*EGR4*	Early Growth Response 4	Schizophrenia 19 and Neuropathy, Congenital Hypomyelinating, 1, Autosomal Recessive
	*SV2C*	Synaptic Vesicle Glycoprotein 2C	Foodborne Botulism and Alcohol-Related Birth Defect
	*GSTT1*	Glutathione S-Transferase Theta 1	Leukoplakia and Senile Cataract
	*EGR1*	Early Growth Response 1	Ischemia and Embryonal Carcinoma
Up regulated	*AQP1*	Aquaporin 1	Blood Group, Colton System and Diabetes Insipidus, Nephrogenic, Autosomal
	*EFEMP1*	EGF Containing Fibulin Extracellular Matrix Protein 1	Doyne Honeycomb Retinal Dystrophy and Inguinal Hernia
	*ABCA8*	ATP Binding Cassette Subfamily A Member 8	Ichthyosis, Congenital, Autosomal Recessive 4B and Autosomal Recessive Congenital Ichthyosis
	*AQP4*	Aquaporin 4	Brain Edema and Neuromyelitis Optica
	*OGN*	Osteoglycin	Inhibited Male Orgasm and Cornea Plana

**Table 3:** Demographical information. PMI, postmortem interval in hours.

Array	Samples	Number of Samples	Male/Female	Age (± SD or Range)	PMI (± SD or Range)
GSE122063	Control	10	5/5	78.6 (8.5)	9 (5–14)
	AD	12	4/8	80.9 (7.4)	8 (4–15)
	VaD	9	5/4	81.4 (10.1)	10 (4–17)
GSE84422	Control	11	5/6	81.7 (12.8)	6.2 (4.3)
	AD	21	7/14	84.8 (9.1)	5.0 (3.3)
GSE118553	Control	21	12/9	69.8 (15.4)	40.4 (24.6)
	AD	38	13/25	82.5 (4.7)	39.4 (20.5)
GSE13162	Control	8	7/4	67 (54–75)	7 (5–14.5)
	FTD	10	4/6	64 (53–72)	7.5 (2–11)

## Supplementary Materials

The following are available online at https://www.mdpi.com/1422-0067/21/6/2050/s1.
